# Linking physical activity to health-related quality of life among middle-aged adults: the mediating roles of resilience and physical exercise self-efficacy

**DOI:** 10.3389/fpubh.2026.1789438

**Published:** 2026-03-30

**Authors:** Qiwei Yang, Binbin Liu, Shengnan Qin, Jiayi Huang, Qingyi Zhang

**Affiliations:** 1 School of Physical Education, Hunan University of Science and Technology, Xiangtan, China; 2 Department of Exercise and Sport Science, Hoseo University, Asan-si, Republic of Korea

**Keywords:** health-related quality of life, middle-aged adults, physical activity, physical exercise self-efficacy, resilience

## Abstract

**Introduction:**

Middle-aged adults increasingly face cumulative health risks and functional challenges, making the maintenance of health-related quality of life (HRQoL) a key public health concern. Although physical activity is consistently associated with better perceived health, less is known about the psychological resources that may help explain this association during midlife. This study examines whether resilience and physical exercise self-efficacy are associated with the link between physical activity and HRQoL among middle-aged adults.

**Methods:**

A cross-sectional survey was conducted among 538 middle-aged adults living in community settings in Hunan Province using a cluster random sampling strategy. Structural equation modelling was used to examine the associations among physical activity, resilience, physical exercise self-efficacy, and HRQoL.

**Results:**

Physical activity was positively associated with resilience, physical exercise self-efficacy, and HRQoL. Both resilience and physical exercise self-efficacy were also positively related to HRQoL. Bootstrap analyses indicated a significant indirect association between physical activity and HRQoL through the joint contribution of resilience and physical exercise self-efficacy.

**Discussion:**

These findings suggest that physical activity is linked to HRQoL not only directly, but also through key psychological resources among middle-aged adults. By highlighting the joint roles of resilience and physical exercise self-efficacy, this study underscores the importance of integrating behavioral engagement and psychological adaptation when examining well-being in midlife.

## 1 Introduction

Globally, population ageing and the rising burden of non-communicable diseases (NCDs) have emerged as persistent challenges for public health systems (1). According to the World Health Organization, NCDs were responsible for approximately 75% of global deaths in 2021, including around 18 million premature deaths before the age of 70 years (2). Among the key lifestyle-related risk factors contributing to the NCD burden, physical inactivity has been identified as a major public health concern. Recent global estimates indicate that nearly one third (31%) of adults worldwide—approximately 1.8 billion people—did not meet the recommended levels of physical activity in 2022 (3). Moreover, the prevalence of insufficient physical activity increased by about 5 percentage points between 2010 and 2022, and, if current trends persist, inactivity levels are projected to rise to approximately 35% by 2030, suggesting that the world is currently off track from meeting the global target to reduce physical inactivity by 2030 (3, 4).

These challenges are particularly salient during midlife, a life-course stage often characterized by accumulated work demands, family responsibilities, and the early emergence of functional limitations (5). In this context, conventional health indicators focused solely on morbidity and mortality provide an incomplete account of population health. Increasingly, HRQoL has been recognized as a critical outcome in global health research, as it is commonly defined as individuals’ subjective perceptions of their physical health, psychological well-being, and social functioning as influenced by health status (6). Within health outcomes research, HRQoL has been conceptualized as a multidimensional construct encompassing physical functioning, mental health, vitality, and social role performance (7). Understanding factors associated with HRQoL among middle-aged adults is therefore essential for informing strategies aimed at promoting healthy ageing and mitigating the long-term societal burden of chronic disease (8).

Physical activity has consistently been identified as a critical health-promoting behavior across the lifespan, with well-documented benefits for both physical and psychological well-being (9, 10). Empirical studies have shown that regular engagement in physical activity is associated not only with reduced risk of chronic diseases, improved cardiovascular and musculoskeletal function, and better metabolic health, but also with enhanced mental health, life satisfaction, and overall HRQoL (11–13). Evidence from both general adult populations and clinical samples indicates that individuals who participate in higher levels of leisure-time or structured physical activity report higher scores on measures of HRQoL, including physical functioning, vitality, and emotional well-being (14, 15). These associations have been observed globally, across different age groups and health conditions, suggesting that physical activity constitutes a broadly relevant and modifiable determinant of perceived health status (16). However, emerging research suggests that the relationship between physical activity and HRQoL may not be purely behavioral or physiological in nature. Instead, psychological resources, such as individuals’ perceived capability to engage in exercise and their capacity to adapt positively to stress and adversity, may play an important role in translating physical activity into enhanced quality of life (17). This shift toward integrating behavioral and psychological perspectives highlights the need for a more nuanced understanding of how and why physical activity influences HRQoL, particularly among middle-aged adults facing accumulating life demands and health risks.

Although numerous studies and systematic reviews have documented a positive association between physical activity and HRQoL across the adult lifespan, important gaps remain in our understanding of the psychological processes that underpin this relationship. Evidence synthesized in reviews and meta-analyses indicates that higher levels of physical activity are consistently associated with better HRQoL and well-being in general and older adult populations (18). For example, intervention studies and randomized controlled trials have demonstrated direct benefits of physical activity on multiple domains of HRQoL (19, 20), and comprehensive reviews have concluded that these effects are generally robust across settings. However, these bodies of work also note limitations in the depth of evidence on causal mechanisms and psychosocial mediators, particularly in non-clinical, non-older adult samples. Specifically, research on psychological mediators such as self-efficacy has provided moderate support for effects on related health outcomes like mental health and psychological distress, but much of this evidence comes from studies focusing on depression, anxiety, or affective states rather than HRQoL as a multidimensional construct (21, 22). Similarly, resilience has been examined as a correlate of well-being and adaptive functioning, but with most studies focusing on younger or older adult groups and outcomes centered on psychological adjustment rather than HRQoL per se.

In addition to these limitations, age-specific variability in the physical activity–HRQoL association has been observed, suggesting that the strength and nature of these relationships may differ across the life course. Cross-sectional analyses in adult samples, have reported differential associations between physical activity and HRQoL across age groups, which implies that life-stage contexts may shape how physical activity relates to subjective health outcomes (23, 24). Beyond individual life-stage differences, emerging evidence suggests that organizational and environmental supports also shape physical activity engagement and its health benefits. Recent research indicates that workplace investments in physical activity infrastructure, such as on-site gym facilities, can promote sustained participation in physical activity and improve physical and psychological health, particularly when institutional support is aligned with individual health behaviors (25). These contextual influences may be especially relevant for middle-aged adults, whose opportunities for engaging in health-promoting behaviors are often constrained by work demands and time pressures.

Despite growing recognition of age- and context-related influences, few studies have examined middle-aged adults as a distinct group or empirically investigated how psychological resources jointly contribute to the physical activity–HRQoL link in this life stage. This gap is notable given that midlife is a period characterized by increasing functional demands and psychosocial stressors, and that psychological resources such as self-efficacy and resilience have been independently linked to both health behaviors and perceived well-being (26, 27). Taken together, while the relevance of physical activity and psychological factors to HRQoL is well supported, there remains a paucity of research integrating multiple psychological constructs into a coherent explanatory framework for middle-aged adults. Addressing this gap can clarify how behavioral engagement and psychological adaptation jointly relate to subjective quality of life, and can inform interventions that target both behavioral and psychosocial determinants of well-being.

Building on the gaps identified in prior research, the present study aims to examine how physical activity is associated with HRQoL among middle-aged adults, with a focus on the mediating roles of resilience and physical exercise self-efficacy. Specifically, this study investigates (1) the direct association between physical activity and HRQoL, (2) the relationships between physical activity and key psychological resources, including resilience and Physical exercise self-efficacy, and (3) the extent to which these psychological factors jointly mediate the association between physical activity and HRQoL. By focusing on middle-aged adults as a distinct life-stage group and integrating multiple psychological constructs within a single framework, the study seeks to provide a more comprehensive understanding of the behavioral and psychosocial mechanisms that may contribute to well-being in midlife.

This study makes several important contributions to the literature on physical activity and HRQoL. First, it addresses a methodological gap by concentrating specifically on middle-aged adults, a population that has received relatively limited attention compared with younger or older adults in previous physical activity–HRQoL research. Second, the study integrates two psychological constructs-resilience and physical exercise self-efficacy-within a single explanatory model, responding to calls from prior research for a more nuanced understanding of the psychosocial mechanisms linking physical activity to HRQoL (28, 29). Third, the findings have practical implications for health promotion, suggesting that interventions designed to enhance physical activity may be more effective if they simultaneously target the development of psychological resources, such as confidence in exercise ability and adaptive coping skills, thereby contributing to improved subjective health and well-being in midlife.

The paper is organized into the following sections: Section 2 outlines the hypotheses and conceptual models. Section 3 details the methods used for data collection and analysis. Section 4 presents the results of the data analysis and tests the proposed hypotheses. Section 5 provides a discussion, including theoretical contributions, practical implications, and limitations, along with suggestions for future research. Finally, Section 6 offers the conclusion.

## 2 Literature review and hypothesis development

### 2.1 Physical activity, resilience, and physical exercise self-efficacy

Resilience is commonly conceptualized as a dynamic process reflecting individuals’ capacity to adapt positively to stress, adversity, and changing life demands. Early theoretical work defined resilience as a process of positive adaptation despite significant adversity, while developmental research further emphasized resilience as “ordinary adaptive processes” that enable individuals to maintain functioning under stress (30). In adulthood, resilience has been increasingly viewed as a modifiable psychological resource rather than a fixed personality trait, shaped by both environmental exposures and behavioral experiences (31). This perspective is also consistent with clinical conceptualizations of resilience as a multidimensional construct involving emotional regulation, coping flexibility, and adaptive functioning (32).

Empirical evidence suggests that individuals who engage in regular physical activity tend to report higher levels of resilience and stress tolerance (33). Cross-sectional and longitudinal studies have shown that physical activity is associated with lower perceived stress, better emotional regulation, and enhanced coping capacity, all of which are considered core components of resilience (34, 35). From a behavioral standpoint, physical activity repeatedly exposes individuals to manageable physical challenges, effortful engagement, and recovery processes, which may strengthen adaptive responses to stress over time (36). In addition, exercise-related improvements in mood regulation and physiological stress responses have been proposed as mechanisms linking physical activity to greater resilience (37). Evidence from adult samples further supports this association. Studies have reported that physically active adults demonstrate greater resilience when facing work-related stressors and daily life challenges (38). Accordingly, we propose the following hypotheses:

*Hypothesis 1 (H1)*: Physical activity is positively associated with resilience.

Physical exercise self-efficacy refers to individuals’ beliefs in their capability to successfully initiate and maintain exercise behavior, even in the presence of barriers such as fatigue, time constraints, or competing responsibilities. The concept of self-efficacy originates from social cognitive theory, which defines self-efficacy as individuals’ beliefs about their capability to organize and execute courses of action required to manage prospective situations (39). Within this theoretical framework, self-efficacy is considered a central determinant of health behavior, and mastery experiences derived from actual behavioral engagement are viewed as its most influential source (40).

In the context of physical activity, physical exercise self-efficacy represents a domain-specific form of efficacy belief referring to confidence in one’s ability to engage in and maintain regular exercise behavior (41). A substantial body of research has demonstrated that participation in physical activity is positively associated with physical exercise self-efficacy. Individuals who engage more frequently in physical activity tend to report greater confidence in their ability to perform exercise tasks, regulate effort, and sustain activity over time (42). These associations have been observed across diverse age groups, including adults and middle-aged populations, suggesting that accumulated exercise experience reinforces perceptions of competence and control (43, 44). In addition, longitudinal studies indicate that changes in physical activity are accompanied by corresponding changes in physical exercise self-efficacy, further supporting the close linkage between behavioral engagement and efficacy beliefs (45). Accordingly, we propose the following hypotheses:

*Hypothesis 2 (H2)*: Physical activity is positively associated with physical exercise self-efficacy.

Resilience and self-efficacy represent related but distinct psychological resources. Resilience reflects individuals’ capacity to adapt to stress and recover from adversity, whereas self-efficacy refers to beliefs about one’s capability to execute actions required to manage specific demands (46). Within social cognitive theory, efficacy beliefs influence how individuals approach challenges, regulate effort, and persist in the face of difficulties (47). Importantly, self-efficacy is inherently domain-specific, and physical exercise self-efficacy denotes confidence in one’s ability to initiate and maintain regular exercise behavior, particularly when facing common barriers such as fatigue, time constraints, or competing responsibilities (48, 49).

Empirical research has consistently documented positive associations between resilience and self-efficacy at a general level, suggesting that individuals with greater adaptive capacity tend to report stronger beliefs in their ability to cope with challenges across domains (50, 51). From a social cognitive perspective, resilient individuals are more likely to interpret difficulties as manageable and temporary, which may support the development of stronger efficacy beliefs through successful coping experiences (52). Although direct empirical evidence linking resilience specifically to physical exercise self-efficacy remains relatively limited, studies in physical activity contexts indicate that resilience may facilitate sustained engagement in exercise by helping individuals persist despite setbacks such as physical discomfort or motivational fluctuations (53). Repeated experiences of overcoming such barriers may, in turn, strengthen confidence in one’s ability to engage in and maintain physical exercise. Accordingly, we propose the following hypotheses:

*Hypothesis 3 (H3)*: Resilience is positively associated with physical exercise self-efficacy.

### 2.2 Resilience, physical exercise self-efficacy, and HRQoL

A growing body of empirical research has demonstrated that resilience is positively associated with health-related quality of life across diverse populations. Studies conducted in community-based adult samples as well as in populations facing health-related challenges have consistently found that individuals with higher levels of resilience report better perceived physical functioning, psychological well-being, and overall quality of life (54, 55). These findings suggest that resilience is closely linked to how individuals perceive and evaluate their health status in daily life. Theoretically, resilience is thought to contribute to HRQoL through its association with adaptive coping processes and emotional regulation. Developmental and health psychology perspectives suggest that resilient individuals are better able to mobilize psychological resources, regulate emotional responses, and maintain functioning when encountering stressors (56). Individuals with higher resilience are more likely to maintain psychological stability under stress, regulate negative emotional responses, and adopt constructive appraisals of health-related difficulties, all of which are central to subjective evaluations of functioning and well-being (57, 58). Rather than eliminating stressors, resilience appears to shape how individuals interpret and respond to ongoing demands, thereby influencing perceived quality of life. Accordingly, we propose the following hypothesis:

*Hypothesis 4 (H4)*: Resilience is positively associated with HRQoL.

Research in health psychology has consistently demonstrated a positive association between self-efficacy and HRQoL. Across a wide range of populations, individuals with higher self-efficacy tend to report better perceived health, functional status, and overall quality of life (59, 60). This pattern has been observed in both clinical and non-clinical samples and is supported by evidence from cross-sectional studies as well as meta-analytic findings, suggesting a robust and stable relationship between perceived capability in managing health-related demands and HRQoL (61–63).

Early research in chronic disease management also demonstrated that stronger self-efficacy beliefs are associated with improved health behaviors, better symptom management, and higher perceived quality of life (61). Building on this broader evidence, subsequent research has focused on domain-specific forms of self-efficacy, particularly physical exercise self-efficacy. As a context-specific belief related to individuals’ confidence in engaging in regular exercise, Physical exercise self-efficacy has been shown to be positively associated with HRQoL, especially in terms of perceived physical functioning and overall health among adult populations. Empirical studies among middle-aged and older adults, as well as those involving general and clinical samples, consistently indicate that individuals who report higher physical exercise self-efficacy also tend to report better HRQoL (64–66). Accordingly, we propose the following hypotheses:

*Hypothesis 5 (H5)*: Physical exercise self-efficacy is positively associated with HRQoL.

### 2.3 Physical activity and HRQoL

A large body of research has consistently documented a positive association between physical activity and HRQoL. Early studies in general adult populations showed that higher levels of leisure-time physical activity were associated with better perceived physical health, psychological well-being, and overall quality of life, establishing physical activity as an important correlate of HRQoL (67, 68). These findings are consistent with broader public health frameworks suggesting that regular physical activity contributes to improvements in both physical functioning and mental well-being, which are central dimensions of HRQoL (69). Subsequent research has extended these findings across different life stages, indicating that the positive association between physical activity and HRQoL is evident not only in adults but also in younger populations, suggesting a broadly applicable relationship between habitual physical activity and perceived health status (70, 71).

Evidence from clinical populations further reinforces this association. Studies focusing on individuals with chronic conditions have demonstrated that variations in physical activity levels are accompanied by corresponding differences in HRQoL, highlighting the close linkage between physical activity engagement and perceived health and functioning (72). Moreover, recent meta-analytic evidence has provided robust support for a positive association between physical activity and HRQoL across diverse samples and study contexts, underscoring the stability of this relationship (73, 74). Accordingly, we propose the following hypotheses:

*Hypothesis 6 (H6)*: Physical activity is positively associated with HRQoL among middle-aged adults.

### 2.4 Mediation effects

Previous research has shown that physical activity is associated with HRQoL, and that this association is closely linked to various psychological resources. Existing studies have independently demonstrated that physical activity is positively associated with resilience and physical exercise self-efficacy, and that each of these psychological factors is, in turn, associated with HRQoL (37, 64). Together, this body of evidence suggests that the relationship between physical activity and HRQoL may be partly explained by multiple psychological correlates rather than by a single factor alone.

Although prior research has rarely examined resilience and physical exercise self-efficacy simultaneously within a single analytical framework, both constructs have been identified as relevant psychological correlates of physical activity and perceived health outcomes. Resilience reflects individuals’ adaptive capacity in the face of stress and health-related challenges (53), while physical physical exercise self-efficacy reflects confidence in engaging in and maintaining exercise behavior (75). Examining these psychological factors together allows for a more comprehensive assessment of how physical activity is associated with HRQoL. Based on existing empirical findings linking physical activity with resilience and physical exercise self-efficacy, as well as linking these psychological factors with HRQoL, we propose the following hypothesis:

*Hypothesis 7 (H7)*: Resilience and physical exercise self-efficacy jointly mediate the association between physical activity and HRQoL.

**Figure 1:**
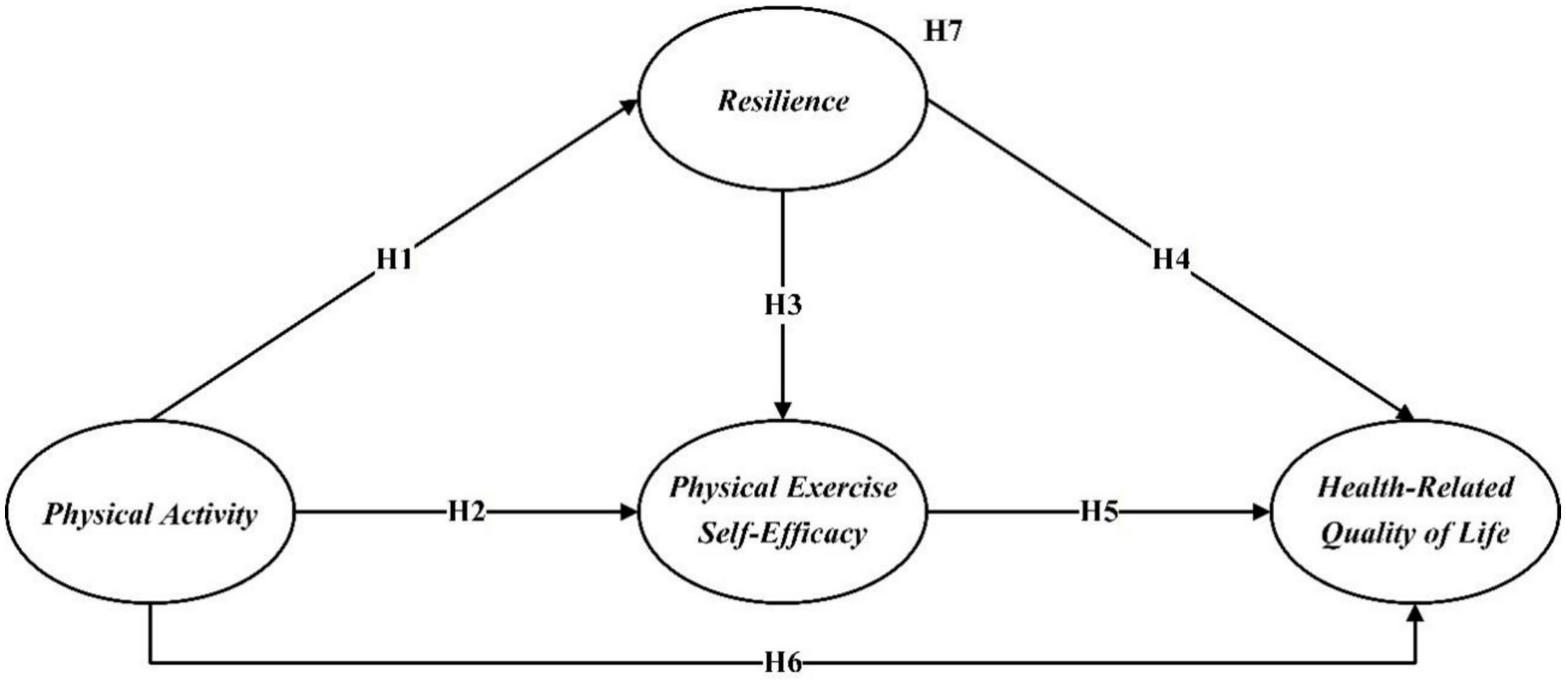
Proposed research model.

Figure 1 presents an overview of all hypothesized relationships.

## 3 Methodology

### 3.1 Participants and procedures

This study surveyed middle-aged adults living in community settings in Hunan Province to examine physical activity behaviors and their associations with resilience, physical exercise self-efficacy, and HRQoL. Participants were recruited from community sports environments as well as surrounding residential areas, allowing the sample to include individuals with varying levels of engagement in physical activity. Hunan Province is a densely populated region with diverse urban–rural communities and well-established public sports facilities, making it an appropriate setting for examining physical activity patterns among middle-aged adults in China. To enhance the diversity of the sample, the survey covered multiple cities across different geographic areas of the province, and participants were recruited from both community sports facilities and nearby residential communities. This approach helped include middle-aged adults from a range of community contexts and levels of physical activity participation. Therefore, although the sample was not designed to be nationally representative, it can be considered reasonably reflective of community-dwelling middle-aged adults in Hunan Province and provides a useful basis for understanding physical activity-related behaviors among community-dwelling middle-aged adults in China.

A cluster random sampling strategy was employed during July–August 2025. Taking the provincial capital, Changsha, as the center, the province was divided into ten geographic zones: east, west, south, north, northwest, northeast, southwest, south-central, central, and the central zone where Changsha is located. One city was randomly selected from each zone, resulting in the inclusion of Xiangtan, Huaihua, Yongzhou, Yiyang, Jishou, Changsha, Yueyang, Shaoyang, Chenzhou, and Loudi. In each selected city, one community sports facility and its surrounding residential community were randomly chosen as survey sites. Middle-aged residents were approached in these locations and invited to participate regardless of their habitual physical activity level.

A total of 600 questionnaires were distributed, and 538 valid responses were collected, yielding an effective response rate of 89.67%. The final sample included 47.4% men and 52.6% women. Most participants were aged 45–60 years (80.6% in total). In terms of educational attainment, 26.4% had completed junior middle school or below, 39.0% had completed senior high school or secondary vocational education, and 34.6% held a college degree or above. Regarding health-related behaviors, 57.2% reported never smoking, whereas 24.2% were current smokers. In addition, 43.1% reported never drinking alcohol, 37.4% drank occasionally, and 19.5% drank often or every day. Detailed sociodemographic characteristics of the participants are presented in Table 1.

**Table 1:** Sociodemographic characteristics of the participants (*N* = 538).

Variable	Category	%
Gender	Male	47.4
	Female	52.6
Age	40–44	19.3
	45–49	34.9
	50–54	22.7
	55–60	23.0
Education level	Junior middle school or below	26.4
	Senior high school or secondary vocational school	39.0
	College diploma or bachelor’s degree	25.7
	Master’s degree or doctorate	8.9
Smoking status	Never smoke	57.2
	Used to smoke but have quit	18.6
	Currently smoke	24.2
Alcohol consumption	Never	43.1
	Occasionally	37.4
	Often	11.3
	Every day	8.2

### 3.2 Instruments

The questionnaire comprised five sections. The first section collected participants’ sociodemographic information, including gender, age, education level, smoking status, and alcohol consumption.

The second section assessed physical activity using three items adapted from the Physical Activity Rating Scale (76). The three items measured exercise intensity, exercise duration, and exercise frequency during the past month, respectively: (1) “What is the intensity of physical activity that you usually participate in during the past month?”, (2) “How long do you spend in each physical activity session?”, and (3) “How often do you do physical activity every month/week?” In the present study, each item was assessed using a five-category response format scored from 1 to 5, with higher scores indicating higher intensity, longer duration, and more frequent participation in physical activity. These three items were selected because they capture the core dimensions of physical activity behavior while remaining concise and practical for use in large-scale community surveys.

The third section measured psychological resilience using the full six-item version of the Brief Resilience Scale (BRS) (77). All six original items were retained in the present study. The items were: “I tend to bounce back quickly after hard times,” “I have a hard time making it through stressful events,” “It does not take me long to recover from a stressful event,” “It is hard for me to snap back when something bad happens,” “I usually come through difficult times with little trouble,” and “I tend to take a long time to get over setbacks in my life.” Items 2, 4, and 6 were reverse coded. Responses were recorded on a five-point Likert-type scale ranging from 1 (Strongly Disagree) to 5 (Strongly Agree), with higher scores indicating greater resilience.

The fourth section assessed physical exercise self-efficacy using the full five-item brief version of the Self-Efficacy for Exercise (SEE) Scale (78). All five original items from this brief version were retained: “Feeling pain when exercising,” “Did not enjoy the exercise,” “Too busy for exercise,” “Feeling tired,” and “Bad mood.” Responses were rated on a five-point Likert-type scale ranging from 1 (Strongly Disagree) to 5 (Strongly Agree). Because these items describe barriers to exercise, the items were reverse coded so that higher scores reflected greater physical exercise self-efficacy.

The fifth section evaluated HRQoL using the 10-item version of the PROMIS Global Health Scale (79). All 10 original items were retained, including global evaluations of general health, quality of life, physical health, mental health, social satisfaction, physical functioning, pain, fatigue, social role performance, and emotional problems. In the present study, to maintain consistency in questionnaire administration, the response options were standardized into a five-point format across items, with higher scores indicating better health-related quality of life after reverse coding where necessary.

### 3.3 Data analysis

Data were analyzed using structural equation modelling (SEM) in AMOS 23.0 to test the proposed relationships among physical activity, psychological resilience, physical exercise self-efficacy, and HRQoL. Models were estimated with maximum likelihood, with a two-stage procedure in which the measurement model was first validated (reliability and convergent/discriminant validity), followed by evaluation of the structural model, including overall fit, standardized path coefficients, and mediated effects. For each proposed research hypothesis, the corresponding null hypothesis assumed that no statistically significant relationship existed between the relevant variables.

To examine whether common method variance (CMV) posed a serious concern given the use of self-report measures, we followed the procedure suggested by Mossholder, Bennett (80) and compared a single-factor CMV model with the hypothesized multifactor measurement model. The single-factor model showed very poor fit, *χ*^2^(275) = 2937.657, *p* < 0.001, whereas the measurement model provided a much better fit to the data, *χ*^2^(246) = 304.344, *p* < 0.01. This substantial difference in model fit indicates that CMV is unlikely to undermine the validity of the study’s results.

## 4 Results

### 4.1 Measurement model

The measurement model was examined by means of confirmatory factor analysis (CFA) in AMOS 23.0. As reported in Table 2, all latent variables demonstrated satisfactory internal consistency, with Cronbach’s *α* coefficients above 0.80, exceeding the commonly recommended threshold for acceptable reliability. In addition, each construct showed an average variance extracted (AVE) greater than 0.50 and composite reliability (CR) values exceeding 0.80, which provided evidence of adequate convergent validity. The standardized factor loadings obtained from the CFA ranged from 0.727 to 0.814 (Table 2), further confirming that the indicators appropriately represented their underlying constructs. Discriminant validity was assessed using the Fornell–Larcker criterion: for each construct, the square root of its AVE was larger than its correlations with the remaining constructs (Table 3), indicating that the constructs were empirically distinct from one another.

**Table 2:** Measurement model.

Items	Factor loadings	Cronbach’s *α*	CR	AVE
Physical activity (PA)		0.821	0.820	0.604
PA1	0.790			
PA2	0.761			
PA3	0.780			
Resilience (RE)		0.911	0.912	0.632
RE1	0.790			
RE2	0.782			
RE3	0.801			
RE4	0.792			
RE5	0.796			
RE6	0.808			
Physical exercise self-efficacy (PES)		0.893	0.895	0.629
PES1	0.795			
PES2	0.806			
PES3	0.755			
PES4	0.814			
PES5	0.795			
Health-related quality of life (HRQoL)		0.927	0.927	0.559
HRQoL1	0.743			
HRQoL2	0.756			
HRQoL3	0.744			
HRQoL4	0.757			
HRQoL5	0.727			
HRQoL6	0.745			
HRQoL7	0.748			
HRQoL8	0.744			
HRQoL9	0.768			
HRQoL10	0.743			

**Table 3:** Correlations and discriminant validity.

Construct	PA	RE	PES	HRQoL
PA	(0.777)			
RE	0.498**	(0.795)		
PES	0.538**	0.480**	(0.793)	
HRQoL	0.453**	0.411**	0.547**	(0.748)

Diagonal entries represent the square roots of AVE; the off-diagonal entries indicate Pearson correlations between the latent constructs. All correlations are significant at *p* < 0.01.

### 4.2 Structural model

**Figure 2:**
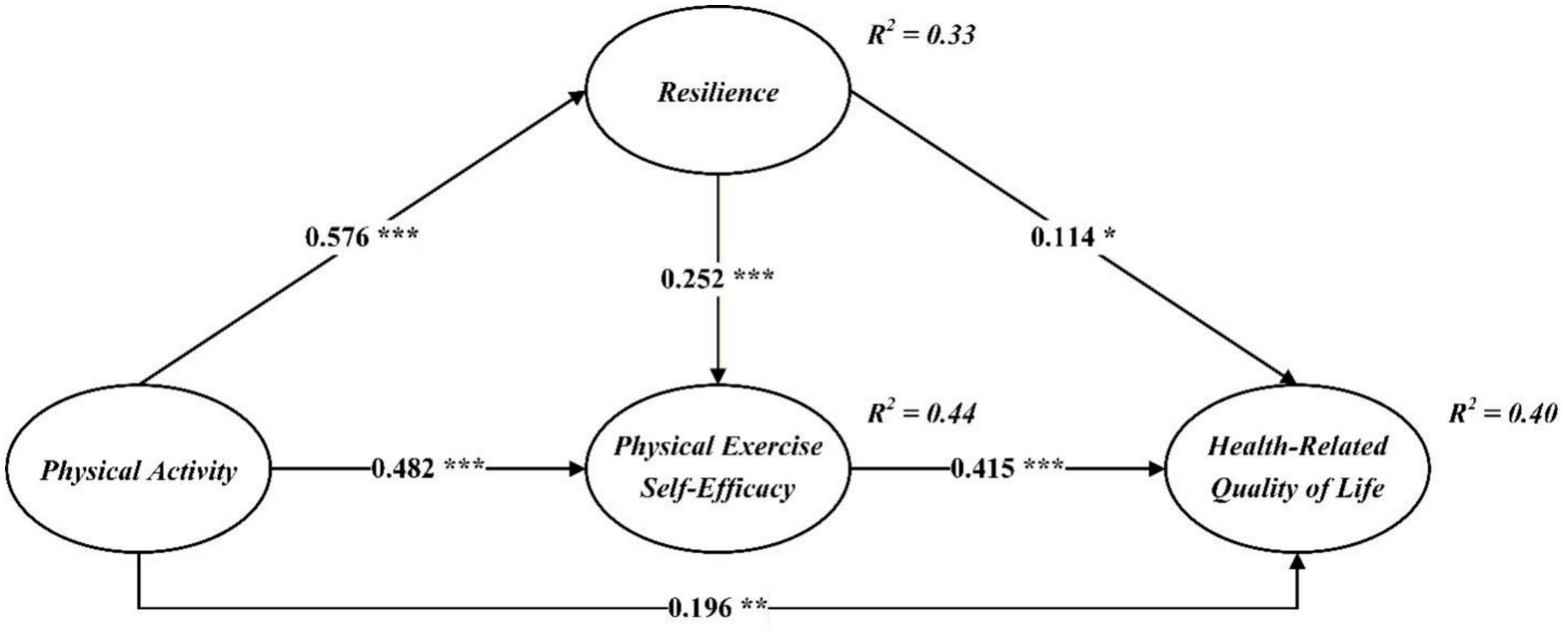
Estimated structural model with standardized path coefficients. The values displayed on the paths are standardized coefficients (*β*), with positive values indicating positive associations among variables. *R*^2^ values represent the proportion of variance explained in each endogenous variable. The structural model was estimated without the inclusion of additional sociodemographic control variables. *p < 0.05, **p < 0.01, ***p < 0.001.

Once the adequacy of the measurement model had been confirmed, we proceeded to estimate the structural model in AMOS 23. Using 5,000 bootstrap samples, the CFA-based fit indices suggested that the model provided an excellent representation of the data (*χ*^2^/df = 1.273, GFI = 0.955, AGFI = 0.945, NFI = 0.961, CFI = 0.992, RMSEA = 0.021). Descriptive zero-order Pearson correlations among the main study variables are presented in Table 3, and the standardized path estimates for the hypothesized model are depicted in Figure 2. The coefficients shown in Figure 2 are standardized path coefficients, which indicate the strength and direction of the associations among the study variables in the structural model.

As shown in Figure 2, physical activity was positively associated with resilience (*β* = 0.576, *p* < 0.001) and physical exercise self-efficacy (*β* = 0.482, *p* < 0.001), thereby supporting H1 and H2. In addition, resilience was positively linked to physical exercise self-efficacy (*β* = 0.252, *p* < 0.001) and to HRQoL (*β* = 0.114, *p* < 0.05), lending support to H3 and H4. Physical exercise self-efficacy, in turn, had a substantial positive effect on HRQoL (*β* = 0.415, *p* < 0.001), in line with H5. Moreover, physical activity itself exerted a significant positive influence on HRQoL among middle-aged adults (*β* = 0.196, *p* < 0.01), thus confirming H6.

We further examined the mediating mechanisms using bias-corrected bootstrap procedures with 5,000 resamples and 95% confidence intervals (Table 4). The results indicated that physical activity exerted a significant indirect effect on HRQoL via resilience and physical exercise self-efficacy, with an indirect coefficient of 0.326 (SE = 0.046, 95% CI [0.239, 0.423], *p* < 0.001). This pattern of findings provides empirical support for H7.

**Table 4:** Bootstrapped indirect effect.

Path	Point estimate	Product of coefficients		Bootstrapping		
				Bias-corrected 95% CI		*p* (two-tailed)
		SE	Z	Lower	Upper	
PA → HRQoL	0.326	0.046	7.087	0.239	0.423	*p* < 0.001

## 5 Discussion

### 5.1 Theoretical contributions

This study makes several theoretical contributions to the literature on physical activity, psychological resources, and HRQoL, particularly in the context of middle-aged adults.

This study contributes to the existing literature on physical activity and HRQoL by extending prior evidence to a middle-aged population within a unified analytical framework. Previous studies and reviews have consistently reported positive associations between physical activity and HRQoL across adult and older populations (28, 68). However, much of this work has either focused on older adults or examined broad adult samples without explicitly considering the midlife stage as a distinct period in the life course. By concentrating on middle-aged adults, the present study adds to the literature by demonstrating that the association between physical activity and HRQoL remains salient during midlife, a stage characterized by increasing health risks and cumulative role demands. In doing so, this study responds to calls for greater attention to age- and life-stage specificity in health behavior research.

A second theoretical contribution lies in the integration of psychological resources into the physical activity and HRQoL framework. Prior research has established that psychological factors such as resilience and self-efficacy are independently associated with physical activity participation and health-related outcomes (17, 66). Studies have also shown that resilience is related to well-being and perceived health, while physical exercise self-efficacy is a robust predictor of physical activity engagement and related quality-of-life outcomes (65). Building on this literature, the present study advances theoretical understanding by examining these psychological resources simultaneously, rather than in isolation, thereby offering a more comprehensive account of how behavioral and psychological factors are associated with HRQoL.

Third, this study contributes to self-efficacy theory by emphasizing the relevance of domain-specific efficacy beliefs for HRQoL. Although general self-efficacy has been linked to various indicators of well-being, social cognitive theory posits that behavior-specific efficacy beliefs are more proximal determinants of behavior-related outcomes. Empirical research in exercise psychology has consistently emphasized the importance of domain-specific efficacy beliefs for understanding physical activity behavior. Guided by social cognitive theory, studies suggest that physical exercise self-efficacy represents a more proximal psychological correlate of exercise participation and maintenance than generalized efficacy beliefs, which tend to reflect broader coping confidence across life domains (64). By focusing on physical exercise self-efficacy and linking it directly to HRQoL, this study extends prior work and underscores the theoretical importance of domain specificity when examining psychological correlates of health outcomes.

Finally, the identification of resilience and physical exercise self-efficacy as mediating mechanisms contributes to the literature by integrating complementary theoretical perspectives. Previous studies have examined resilience as a protective factor for mental health and well-being, and self-efficacy as a key mechanism linking physical activity to health outcomes, but these constructs have often been studied separately (49, 53). The present findings suggest that these psychological resources collectively contribute to explaining the association between physical activity and HRQoL. In other words, both resilience and physical exercise self-efficacy appear to represent complementary pathways through which engagement in physical activity is associated with better perceived health-related quality of life. From a theoretical perspective, this pattern is consistent with social cognitive theory and stress-coping perspectives, which emphasize that behavioral engagement can foster both adaptive coping capacities and stronger efficacy beliefs. Physical activity may enhance individuals’ ability to adapt to daily stressors while simultaneously strengthening their confidence in maintaining health-related behaviors. These psychological resources may therefore function as complementary mechanisms linking health-promoting behaviors to subjective health outcomes.

### 5.2 Practical implications

Although the present study is cross-sectional, the pattern of associations among physical activity, resilience, physical exercise self-efficacy and HRQoL offers several insights that may be useful for community practice with middle-aged adults.

The findings suggest that middle-aged adults who are more physically active tend to report greater psychological resilience and better perceived health. When communities or organizations plan sports and fitness provision for this age group, it may therefore be helpful to think beyond vigorous or performance-oriented exercise and focus on activities that are easier to sustain in everyday life. Examples include walking groups in neighborhoods, tai chi or square dancing in community squares, and other moderate-intensity options that can be fitted around work and family responsibilities. Designing spaces and opportunities that feel safe, accessible and socially welcoming may be particularly relevant for those who have not exercised regularly for some time.

At the same time, the study shows that resilience and physical exercise self-efficacy are closely linked both to physical activity and to HRQoL. In practice, this means that arrangements for community physical activity could usefully take into account not only the quantity of exercise, but also how confident and psychologically supported participants feel. Clear, easy-to-understand guidance on how to exercise within one’s physical limits, chances to start with simple movements and gradually build up, and opportunities to share experiences with peers of a similar age may all help middle-aged adults feel more capable and less intimidated by participation. Activities that are enjoyable, manageable, and supportive of gradual engagement may be especially consistent with the associations observed in this study.

The results also indicate that physical exercise self-efficacy is strongly intertwined with HRQoL in this population. For practitioners working in community sports, health education, or primary care, it may therefore be meaningful to pay attention to messages and arrangements that reduce common worries among middle-aged adults, such as concerns about pain, fatigue, or lack of confidence in exercise participation. Providing realistic examples of how people with similar age and health profiles engage in activity, emphasizing small, achievable steps rather than high performance, and acknowledging the constraints of work and caregiving roles may all help align practice with the correlates identified in the data.

Finally, physical activity showed a positive association with HRQoL even when resilience and self-efficacy were considered. For local policymakers and community planners, this pattern underscores the relevance of physical activity as part of a broader picture of midlife well-being. When decisions are made about neighborhood design, community facilities or workplace health policies, it may be useful to consider whether middle-aged residents have convenient, low-cost and socially acceptable options to be active in ways that suit their life stage.

### 5.3 Limitations

This study is subject to several limitations that point to directions for future research. First, the use of cross-sectional data restricts the ability to infer temporal or causal ordering among the variables. Longitudinal designs or experimental approaches would help clarify whether the sequential patterns observed in this study are stable over time. Second, the sample was drawn from middle-aged adults living in community settings within one province of China, which may limit the generalizability of the findings. Although the survey covered multiple cities in Hunan Province, the sample was not designed to be nationally representative. Comparative studies across different cultural contexts, geographical areas, or age groups would be valuable for examining the robustness of the observed associations. Third, all variables were assessed using self-report measures, which may have introduced response-related bias despite the steps taken to examine common method variance. Fourth, the study focused on overall levels of physical activity, whereas future research may benefit from distinguishing between different types, intensities, or contexts of activity to explore whether these characteristics relate differently to resilience, physical exercise self-efficacy, or HRQoL. Incorporating additional psychosocial factors—such as social support or exercise motivation—may also contribute to a more comprehensive understanding of the mechanisms linking physical activity to well-being in middle-aged adults.

## 6 Conclusion

This study examined the associations among physical activity, resilience, physical exercise self-efficacy, and HRQoL among middle-aged adults living in community settings. The findings indicate that individuals who reported higher levels of physical activity also tended to report greater resilience and higher physical exercise self-efficacy, both of which were positively associated with perceived health. Moreover, resilience and physical exercise self-efficacy jointly accounted for a significant portion of the association between physical activity and HRQoL.

Overall, these results highlight a meaningful pattern in which behavioral engagement and psychological resources are interconnected in shaping middle-aged adults’ perceptions of health and well-being. By integrating physical activity with key psychosocial resources within a single analytical framework, this study contributes to a more nuanced understanding of how health-related quality of life is associated with both behavioral and psychological factors during midlife.

## Data Availability

The raw data supporting the conclusions of this article will be made available by the authors, without undue reservation.
